# MK256 is a novel CDK8 inhibitor with potent antitumor activity in AML through downregulation of the STAT pathway

**DOI:** 10.18632/oncotarget.28305

**Published:** 2022-11-02

**Authors:** Jen-Chieh Lee, Shu Liu, Yucheng Wang, You Liang, David M. Jablons

**Affiliations:** ^1^Thoracic Oncology, Department of Medicine, University of California, San Francisco, CA 94143, USA; ^2^Touro University, College of Osteopathic Medicine, Vallejo, CA 94592, USA; ^*^These authors contributed equally to this work

**Keywords:** AML, CDK8, kinase inhibitor, STAT pathway, xenograft

## Abstract

Acute myeloid leukemia (AML) is the most lethal form of AML due to disease relapse. Cyclin dependent kinase 8 (CDK8) is a serine/threonine kinase that belongs to the family of Cyclin-dependent kinases and is an emerging target for the treatment of AML. MK256, a potent, selective, and orally available CDK8 inhibitor was developed to target AML. We sought to examine the anticancer effect of MK256 on AML. In CD34+/CD38- leukemia stem cells, we found that MK256 induced differentiation and maturation. Treatment of MK256 inhibited proliferation of AML cell lines. Further studies of the inhibitory effect suggested that MK256 not only downregulated phosphorylated STAT1(S727) and STAT5(S726), but also lowered mRNA expressions of MCL-1 and CCL2 in AML cell lines. Efficacy of MK256 was shown in MOLM-14 xenograft models, and the inhibitory effect on phosphorylated STAT1(S727) and STAT5(S726) with treatment of MK256 was observed *in vivo*. Pharmacologic dynamics study of MK256 in MOLM-14 xenograft models showed dose-dependent inhibition of the STAT pathway. Both *in vitro* and *in vivo* studies suggested that MK256 could effectively downregulate the STAT pathway. *In vitro* ADME, pharmacological kinetics, and toxicity of MK256 were profiled to evaluate the drug properties of MK256. Our results show that MK256 is a novel CDK8 inhibitor with a desirable efficacy and safety profile and has great potential to be a promising drug candidate for AML through regulating the STAT pathway.

## INTRODUCTION

Acute myeloid leukemia (AML) accounts for about 1% of all cancers and is one of the most common types of leukemia affecting adults, especially after the age of 45 (American Cancer Society, 2021). AML accounted for an estimated 20,240 newly diagnosed cases and about 11,400 deaths in the US in 2021 (American Cancer Society, 2021). Standard treatments include chemotherapy with the combination of cytarabine and an anthracycline drug such as daunorubicin or idarubicin. Appropriate patients can also undergo bone marrow transplantation from matched donors. Targeted drug therapies for AML are available in the form of small molecule inhibitors such as FLT3 inhibitors midostaurin and gilteritinib for patients with mutation in the *FLT3* gene; IDH inhibitors ivosidenib and enasidenib for patients with *IDH1* or *IDH2* mutation, respectively; and Bcl-2 inhibitor venetoclax and hedgehog pathway inhibitor glasdegib for patients with overexpressed bcl-2 lymphoid malignancies. The antibody-drug conjugate gemtuzumba ozogamicin is also available as a targeted drug therapy for CD33-positive AML patients. The targeted therapies can be used either alone or in combination with chemotherapies. Despite numerous treatment options that offer high remission rates for patients with AML, it remains the most lethal form of leukemia due to relapse [1, 2].

Leukemic stem cells (LSCs), different from bulk AML populations, are characterized by quiescent cell cycle status, unlimited potential for self-renewal, and ability to initiate and maintain AML. Since standard chemotherapy drugs have little if any effect on LSCs, they are a recognized contributor to the high recurrent rate of AML [2–7]. LSCs exhibit a CD34+/CD38- phenotype, similar to that of normal human hematopoietic stem cells (HSCs), but are characterized by additional cell surface markers, such as CD123 [8], CD96 [9], CLL-1 [10], TIM-3 [11], CD93 [12] and CD99 [13].

Janus kinase/signal transducers and activators of transcription (JAK/STAT) pathway regulates a wide variety of vital biological processes, including embryogenesis, hematopoiesis, cell proliferation, differentiation, apoptosis, and immunity. There are three key components in this pathway: four Janus kinases (JAK1,2,3 and TYK2), seven STATs (STAT1,2,3,4,5a,5b and 6), and receptors which bind to cytokines and growth factors. Upon the binding of cytokines to receptor, JAK is activated to phosphorylate STATs. Dimerization of the phosphorylated STATs(p-STATs) leads to their relocation from cytosol to nucleus where STATs bind to DNA and initiate transcription of target genes. Dysregulation of the JAK/STAT pathway has been associated with various hematological malignancies [14]. Analysis of clinical samples of AML patients suggested that levels of p-JAK2 were inversely correlated with complete remission rates and overall survival [15]. STAT1, STAT3 and STAT5 are constitutively activated in AML cell lines [16]. Overexpression of STAT3 was found in AML and demonstrated to be an indication of shorter survival and worse clinical outcome for patients [17]. Constitutive STAT5 phosphorylation has also been found in 69% of AML [18]. Particularly, STAT5 has been shown to be required for maintenance and expansion of LSCs [19–21]. Therefore, targeting the STAT pathway may represent a promising therapeutic strategy for AML.

Cyclin dependent kinase 8 (CDK8) is a serine/threonine kinase that belongs to the family of Cyclin-dependent kinases (CDKs), which require the association of their cyclin partners to be active [22]. Different from its counterpart proteins CDK1, 2, 3, 4 and 6 that are involved in cell cycle division, CDK8 is a transcription-regulating kinase that controls gene expression via phosphorylation of the highly unstructured c-terminal domain (CTD) of RNA polymerase II [23, 24]. Partnering with MED12, MED13 and CCNC (cyclin C), CDK8 forms a kinase module as a subunit of the mediator complex to relay transcription signals to its machinery [25, 26]. Through interacting with transcription factors directly, the CDK8 kinase module can regulate signaling pathways such as STAT signaling, transforming growth factor-β (TGF-β), NOTCH-dependent signaling, bone morphogenetic protein (BMP) receptor signaling [24]. Specifically, CDK8 directly phosphorylates S727 in STAT1 in response to IFN-r [27]. CDK8 can also positively regulate phosphorylation of S727 in STAT3, and knockdown of CDK8 leads to reduced levels of STAT3 S727 phosphorylation with INFß induction [27, 28]. SEL120-34A(SEL120), a clinical trial phase I CDK8 inhibitor, has been shown to downregulate phosphorylation level of STAT1 and STAT5 in AML cells lines [29]. CDK8 may downregulate the STAT pathway and could be an attractive and druggable target for AML therapy.

We therefore sought to examine the anticancer effect of MK256, a potent and selective CDK8 inhibitor, on AML. We analyzed how MK256 may exert its effects on LSCs, as well as the regulatory effects of MK256 on the STAT pathway both *in vitro* and *in vivo*. The results suggest that MK256 can downregulate the phosphorylation of STAT1, STAT3 and STAT5 in AML cell lines. We also profiled drug-like properties of MK256, characterized pharmaceutical kinetics and dynamics, and toxicity. MK256 showed great efficacy in AML xenografts both as a single agent and in combination with AML drug venetoclax. Our collective results suggest that MK256 is a potential clinical development candidate as a small molecule therapeutic for patients with AML.

## RESULTS

### MK256 is a potent and selective CDK8 inhibitor

**Figure 1:**
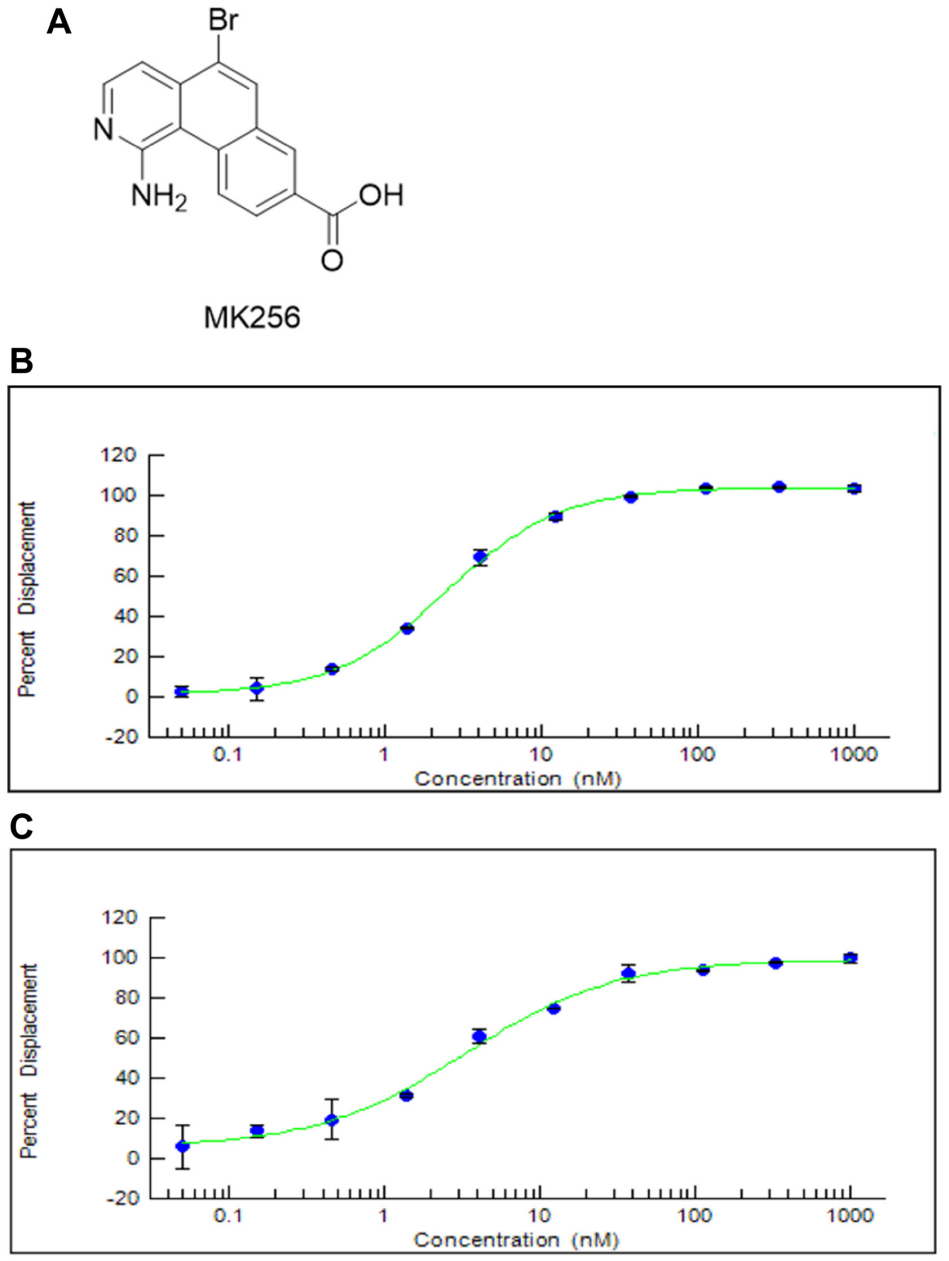
(**A**) Chemical Structure of MK256. (**B**) The inhibition curve of MK256 against CDK8/Cyclin C (IC50: 2.5 nM). (**C**) The inhibition curve of MK256 against CDK19/Cyclin C (IC50: 3.3 nM).

We designed and synthesized a series of tricyclic CDK8 inhibitors through computer-aided drug design (CADD). MK256 (Figure 1A) is among the most potent and selective CDK8 inhibitors in this series. The IC_50_s of MK256 against CDK8/cyclin C (Figure 1B) and CDK19/cyclin C (Figure 1C) were determined as 2.5 nM and 3.3 nM, respectively. To investigate the selectivity of MK256 in the CDK family, we profiled the compound against 13 CDKs at 10 uM. The percentage of kinase inhibition of MK256 for individual CDK at the tested concentration is shown in Table 1. We found that at 10 uM, MK256 showed less than 50% kinase inhibition against both CDK4 and CDK6 but showed more than 90% inhibition against multiple CDKs including CDK1, 5, 9, 13, 14, 17 and 18. To profile the selectivity of MK256 in depth, we tested the compound against the same 13 CDKs at 200 nM. The results showed that none of the CDKs were inhibited significantly by MK256 at 200 nM, except for CDK9. Next, we determined that the IC_50_ of MK256 against CDK9 is 105 nM, which is an approximately 40-fold reduction in comparison to the activity against CDK8.

**Table 1:** Inhibition of CDKs with MK256 at 10 μM and 200 nM

Cyclin dependent kinase	Percentage of inhibition (%)	
	10 μM	200 nM
CDK1/cyclin B	97	34
CDK2/cyclin A	85	7
CDK2/cyclin E1	70	5
CDK3/cyclin E1	82	4
CDK4/cyclin D1	23	2
CDK4/cyclin D3	23	−3
CDK5 (Inactive)	70	7
CDK5/p25	89	2
CDK5/p35	90	7
CDK6/cyclin D1	23	−12
CDK7/cyclin H/MNAT1	54	9
CDK9/cyclin T1	98	82
CDK9/cyclin K	101	76
CDK13/cyclin K	95	27
CDK14 (PFTK1)/cyclin Y	97	23
CDK16 (PCTK1)/cyclin Y	67	1
CDK17/cyclin Y	94	25
CDK18/cyclin Y	98	37

**Figure 2:**
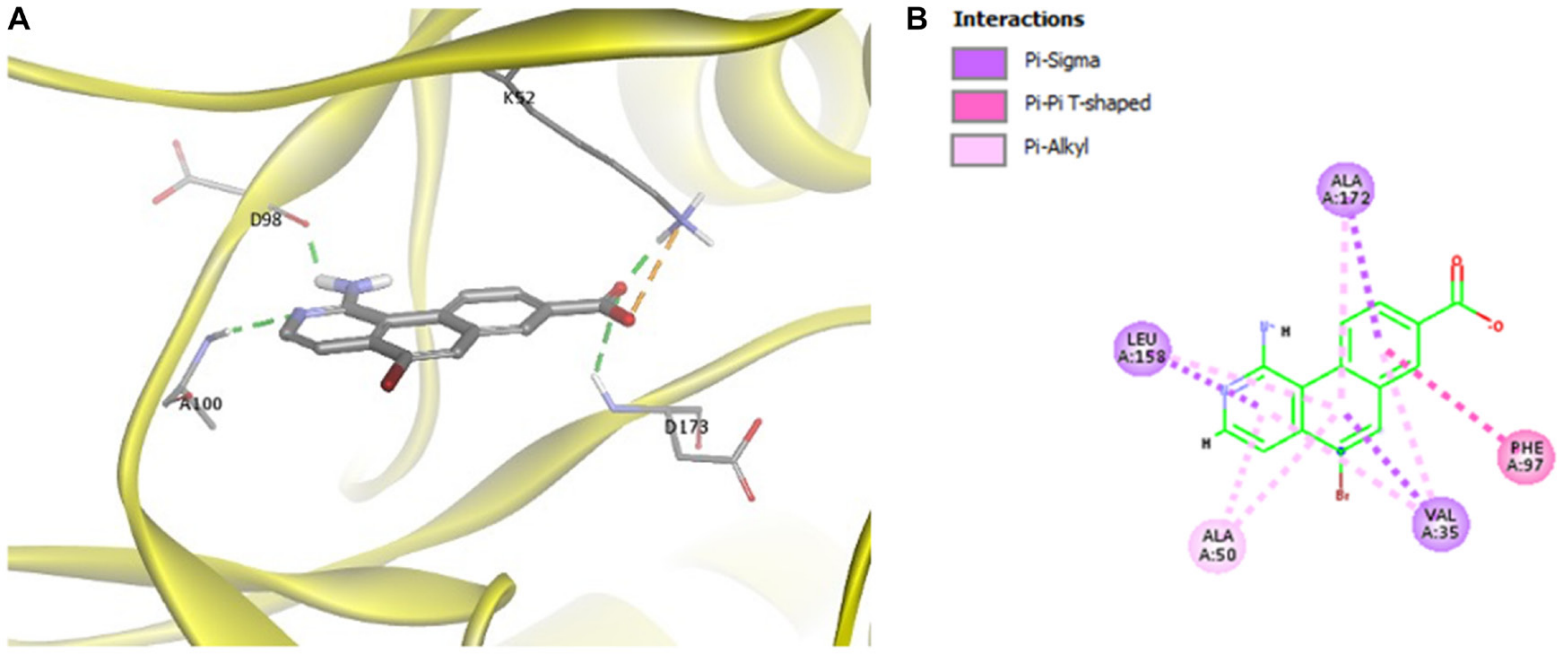
(**A**) Docking of MK256 in the ATP binding pocket of CDK8 (PDB:5HBJ). CDK8 is represented with yellow ribbon. K52, D98, A100, D173 are shown in stick form. H-bond is shown in green dashed line. Ironic interaction is shown in orange dashed line. (**B**) 2D interaction map of MK256 in the ATP binding pocket of CDK8. Interactions of Pi-Sigma, Pi-Pi T-shaped, Pi-Alkyl are shown in purple, pink and light pink dashed line, respectively. Amino acid providing Pi-Sigma, Pi-Pi T-shaped, Pi-Alkyl are shown in purple, pink and light pink dashed line, respectively.

To understand how MK256 bonds in the ATP binding pocket of CDK8, we did docking, followed by molecular dynamics simulation of MK256 in CDK8. Computational simulation suggested that MK256 formed a diverse set of interactions with the surrounding residues inside the ATP pocket (Figure 2A, 2B). Two hydrogen bonds were formed between MK256 and hinge region residues Asp98 and Ala100: 1) the 1-amino group of MK256 acted as a H-bond donor to form one H-bond with the carbonyl of Asp98; 2) nitrogen on the benzo[*h*]isoquinoline ring served as a hydrogen bond acceptor to form the second H-bond with NH of backbone of A100. In addition to the H-bonds in the hinge region, the carbonyl of the carboxylic acid group of MK256 formed one hydrogen bond with Lys52 sitting above the plane of MK256 and another hydrogen bond with Asp173 sitting under the plane of MK256. The hydroxide from the carboxylic acid group of MK256 also formed an electrostatic interaction with Lys52. In addition to H-bonds and electrostatic interactions, MK256 also formed multiple Pi interactions with surrounding hydrophobic residues. MK256 formed Pi-Sigma interaction with Val35, Leu158, Ala172, Pi-Alkyl interaction with Ala50, Val35, Leu158, Ala172, and Pi-Pi T-shaped interaction with Phe97.

### MK256 induces differentiation/maturation in CD34+/CD38- AML TEX cells

**Figure 3:**
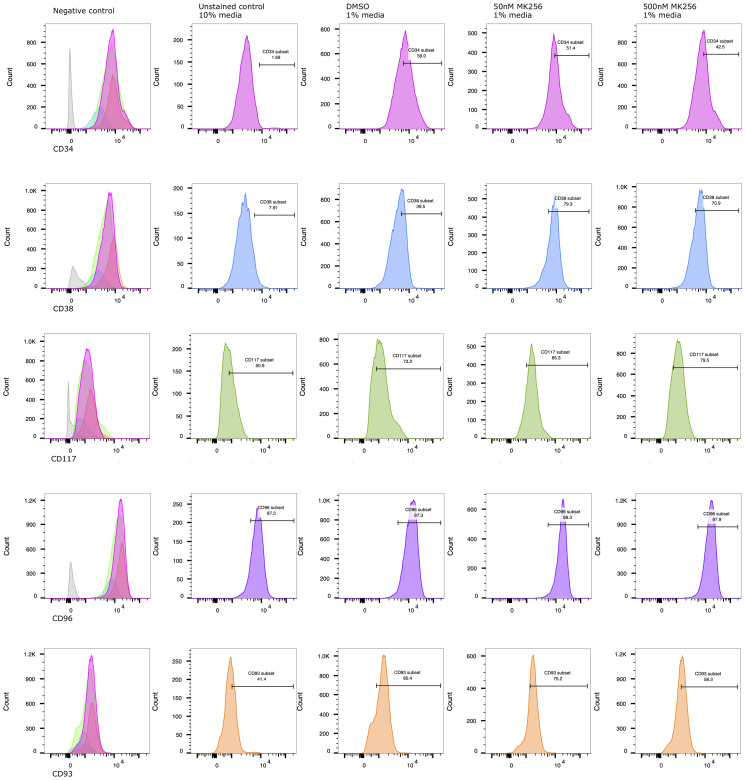
MK256 induces differentiation/maturation in CD34+/CD38- AML TEX cells. At 6th day of treatment, % detection of CD34+, CD93+ populations decreased from 59.0% to 42.5%, 80.4% to 58.3%, respectively. Under the same treatment condition, % detection of CD38+, CD117+ populations increased from 39.5% to 70.9%, 73.2% to 79.5%, respectively. CD96+, as an LSC marker, highly expressed on characteristic AML TEX cells.

After preliminary experiments, we selected 50 nM and 500 nM as the optimal range of MK256 concentrations for the treatment of novel human leukemic cell line (TEX) cells [30], which displayed CD34+/CD38- phenotype. Proliferation and differentiation of TEX was assessed after treatment with MK256 alone (50 nM and 500 nM) and DMSO alone as control. Treatment with MK256 alone at 50 nM slightly induced the differentiation, whereas treatment with higher MK256 concentration 500 nM was much more effective (Figure 3).

To assess if increased differentiation was associated with the treatment, the immunotype of MK256 and DMSO-treated TEX cells was evaluated at 6-day post-treatment by staining with anti-CD34, anti-CD38, anti-CD93, anti-CD96, and anti-CD117. The antibodies selected were to detect common markers of granulocytic and monocytic differentiation. At day 6, we observed a statistically significant decrease in CD34 level, a hematopoietic stem cell (HPSC) marker, in cells treated with MK256. Particularly compared to control cells, cells treated with MK256 at 50 nM and 500 nM led to 13% and 28% (7.6/59≈13% and 16.5/59≈28%) reduction in CD34, respectively.

We also examined CD38 marker, a multi-functional transmembrane glycoprotein that is a lymphocytic receptor and clinical marker for regulation of cytokine release, adhesion, and cellular migration toward sites of inflammation [31]. While most AML blasts showed high CD38 expression without obvious correlation with cytomorphological and genetic characteristics, TEX evidently exhibits genetic changes that express significantly less CD38 [30]. Indeed, FACS analysis previously demonstrated that CD96 is expressed on the majority of CD34+/CD38- AML cells [9], and our data aligned with the findings. The control group in our experiment showed 60% of CD38- TEX phenotype. The approximately 2-fold reduction of CD38 negativity in MK256 treated TEX cells indicated treatment-associated differentiation. On the other hand, the expression of CD96, as an LSC instead of an HPSC marker, showed no difference in TEX with or without the treatment. The maintenance of CD96 presence demonstrated its potential to serve as an LSC-specific therapeutic target [9].

Many genes associated with an undifferentiated “stem-like” phenotype were repressed by the MK256 treatment, including CD93, highly expressed on primary AML specimens, and have been identified as novel therapeutic targets [32]. In our control group, the CD93 population was 80.4%. Although at 50 nM, MK256 decreased CD93 by 6.5%, by when we increased the concentration to 500 nM, MK256 repressed the stem-like marker by approximately 30%.

To further characterize the differentiation of MK256-treated TEX cells, we also assessed anti-CD117, a diagnostic marker for AML to differentiate acute lymphoid leukemia (ALL) [33]. Upon FACS analysis, the CD117 population was 73.2% in the untreated control group, with increases of 16.5% and 8.6% after 50 nM and 500 nM MK256 treatment, respectively.

### Inhibition of AML cell lines by MK256 correlates with the expression levels of STAT1 and STAT5

**Figure 4:**
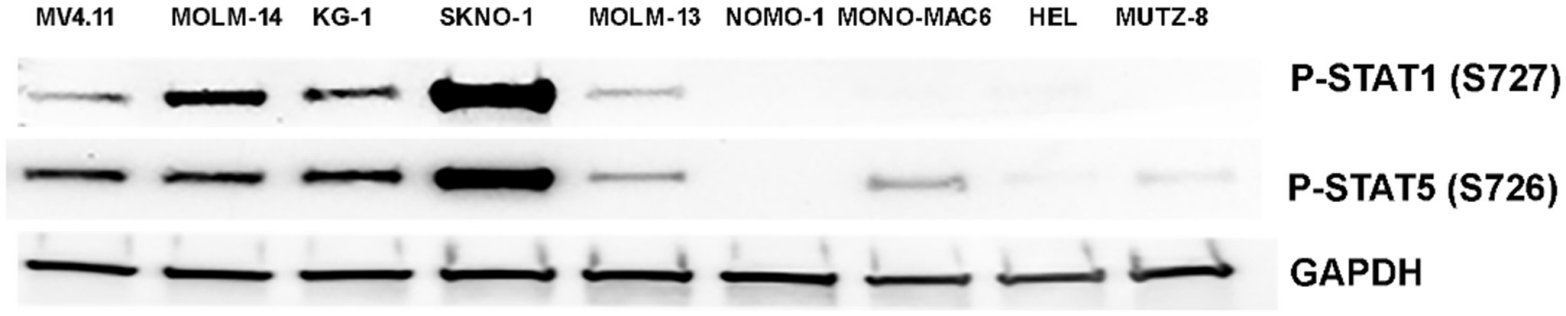
Western of p-STAT1(S727) and p-STAT5(S726) in 9 AML cell lines. Expression level of p-STAT1(S727) and p-STAT5(S726) in MV-4-11, MOLM-14, KG-1, SKNO-1, MOLM-13, NOMO-1, MONO-MAC6, HEL, MUTZ-8 were shown. GAPDH was shown as control.

Next, we evaluated the sensitivity of MK256 against a panel of ten AML cell lines (Table 2). MK256 showed a wide range of sensitivity to the tested AML cell lines, ranging from highly sensitive (<30 nM) to highly resistant (>5 uM). MK256 was the most sensitive to MV-4-11 (IC_50_: 23 nM) and MOLM-14 (IC_50_: 24 nM), both of which harbor mixed lineage leukemia (MLL) fusions. MK256 also showed relatively potent growth inhibition against SKNO-1 (IC_50_: 73 nM) and KG-1 (IC_50_: 80 nM) in this panel. Moderate growth inhibition was observed for SET-2 (IC_50_: 212 nM), NOMO-1(IC_50_: 646 nM), MOLM-13 (IC_50_: 1050 nM) and MONO-MAC-6 (IC_50_: 1258 nM). HEL and MUTZ-8 were among the two most resistant cell lines to MK256 (IC_50_s > 2 uM). After cell line screening, we analyzed at the expression level of p-STAT1(S727) and p-STAT5(S726) in those cell lines via western blotting (Figure 4). The results suggested that MV4.11, MOLM-14, KG-1, and SKNO-1, which were highly sensitive among the cell lines tested, have high expression levels of p-STAT1(S727) and p-STAT5(S726). SKNO-1 had exceptionally high levels of the two p-STATs. MOLM-14 had relatively higher p-STAT1(S727) compared to p-STAT5(S726), whereas MV-4-11 and KG-1 had higher p-STAT5(s726) than p-STAT1(S727). The remaining five less sensitive or resistant cell lines, MOLM-13, NOMO-1, MONO-MAC6, HEL and MUTZ-8, had IC_50_s ranging from 646 nM to 5,559 nM and weak or no expression of p-STAT1(S727) and p-STAT5(S726). These results indicate that sensitivity of MK256 against AML cell lines might be dependent on the expression level of p-STAT1(S727) and p-STAT5(S726).

**Table 2:** Cell proliferation assay of AML cell lines with treatment of MK256

AML cell line	IC_50_ nM (7-day)
MV-4-11	23
MOLM-14	24
SKNO-1	73
KG-1	80
SET-2	212
NOMO-1	646
MOLM-13	1050
MONO-MAC-6	1258
HEL	2700
MUTZ-8	5559

### MK256 downregulates phosphorylation of STAT1 and STAT5 in AML cell lines MV-4-11 and MOLM-14

**Figure 5:**
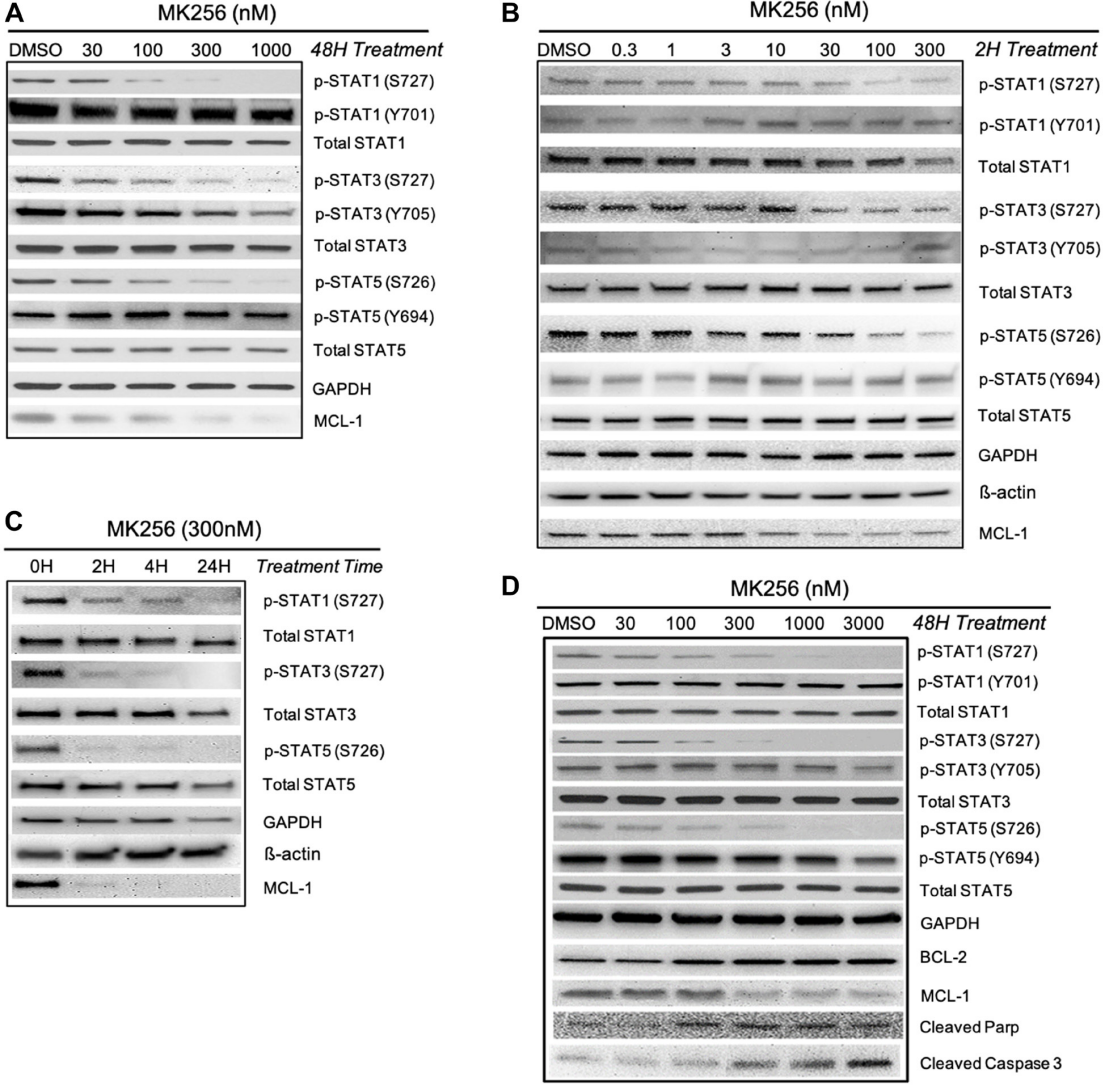
Western blot analysis of MK256 in MV-4-11 and MOLM-14 cell lines. (**A**) 48 hour treatment of MK256 in MV-4-11 cell line. (**B**) 2 hour treatment of MK256 in MOLM-14 cell line. (**C**) Time course study of MK256 (300 nM) in MOLM-14 cell line. Four time points, 0H, 2H, 4H and 24H, were studied. (**D**) 48 hour treatment of MK256 in MOLM-14 cell line.

We were curious to see if MK256, as a CDK8 inhibitor, could regulate the STAT pathway in the highly sensitive AML cell lines. To explore the effect of MK256 on STATs, we chose MV-4-11, the AML cell line our previous experiments showed to be most sensitive to MK256. We challenged MV-4-11 cells with 48 hrs treatment of MK256, with escalating concentrations from 30 nM to 1 uM. The expression level of STAT proteins was illustrated by western blot (Figure 5A). From the immunoblotting of MV-4-11 cells, we observed a trend of reduction in the expression levels of p-STAT1 (S727), p-STAT3(S727), p-STAT5(S726) with increased concentration of MK256. We also observed reduced expression of MCL-1 in a dose-dependent manner after MK256 treatment. At 1 uM of MK256 treatment, p-STAT1(S727) was abrogated completely, whereas MCL-1, p-STAT3(S727), and p-STAT5(S726) remained at minimal levels. Nonetheless, at high concentrations of MK256, the expression levels of p-STAT1(Y701) and p-STAT5(Y694) were both unaffected.

The downregulation of phosphorylated serine on certain STATs was further studied in another AML cell line: MOLM-14. We first examined whether a short period of MK256 treatment in various concentrations would induce any changes in the expression level of p-STATs. At 2-hrs, we observed dose-dependent inhibitions of p-STAT1 (S727), p-STAT3(S727), and p-STAT5(S726), which indicated a fast onset of MK256 (Figure 5B). At 2 hrs, MCL-1 was also downregulated by MK256. Western blot analysis indicated a short onset of CDK8 regulation in the STAT pathway. Next, we increased the treatment time of MK256 from to 4 hrs and 24 hrs to see if prolonged treatment of MK256 would have a more profound inhibitory effect on the phosphorylation of STATs. The results suggested p-levels of STATs were further downregulated in a time-dependent manner (Figure 5C). Next, we escalated the dose of MK256 from 30 nM to 3 mM in the MOLM-14 cell line at a 48 hr time point (Figure 5D). In addition to the STATs panel from the previous experiments, we also introduced apoptosis markers BCL-2, cleaved Parp and cleaved Caspase 3. Western blots showed dose-dependent inhibition of targeted p-STATs and MCL-1 by MK256 in the MOLM-14 cell line, and dose-dependent upregulation of the levels of cleaved Parp and cleaved Caspase 3. However, in the latter, there was unexpected upregulation of expression of BCL-2, suggesting a resistant machinery to cell apoptosis. This may suggest the possible benefit of combining BCL-2 inhibitors with MK256 in treating MOLM-14 cells. In summary, western blot analysis suggests that MK256 can downregulate p-STAT1 (S727), p-STAT3(S727), p-STAT5(S726) in a dose-dependent manner and can trigger apoptosis in two AML cell lines: MV-4-11 and MOLM-14.

### MK256 downregulates MCL-1 and CCL2 mRNA expression in AML cell lines

**Figure 6:**
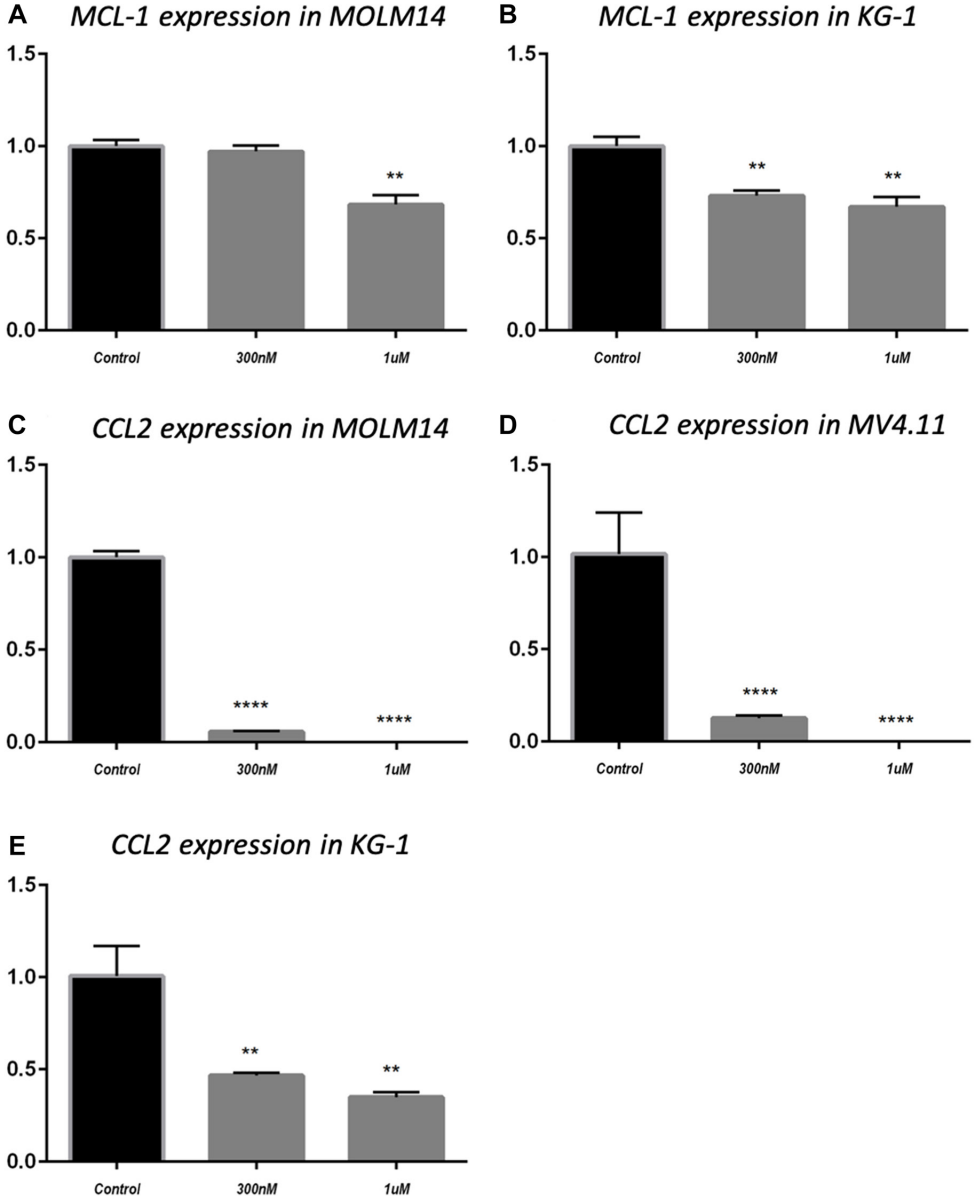
MCL-1 mRNA expression in MOLM14 cell line (**A**) and KG-1 cell line (**B**); CCL2 mRNA expression in MOLM14 cell line (**C**), MV4.11 cell line (**D**) and KG-1 cell line (**E**). ^**^ *P* < 0.01; ^****^ *P* < 0.0001.

BCL-2 family proteins are critical in determining the fate of cells between death and survival [34]. MCL-1 is an anti-apoptotic protein that belongs to BCL-2 family. MCL-1 has been found to be increased in AML and is a target for the disease [35–37]. Having shown that MCL-1 protein level was downregulated by MK256 in MOLM-14 cells and MV4.11 cells under various treatment conditions (Figure 5), we were intrigued to see if MK256 could induce any type of change of MCL-1 at the transcription level. Quantitative real-time RT-PCR was used to determine mRNA levels of MCL-1 in two AML cell lines, MOLM-14 and KG-1. MCL-1 mRNA levels were plotted as fold change over basal levels. The MCL-1 mRNA level barely changed upon treatment with MK256 at 300 nM in MOLM-14 cells; at 1 uM, the MCL-1 mRNA level was downregulated by ~ 30% (Figure 6A). In KG-1 cells, both 300 nM and 1 uM of MK256 decreased the mRNA level of MK256 to 25% and 30%, respectively (Figure 6B).

In addition to MCL-1, we evaluated the effect of treatment of MK256 on the mRNA level of the C-C motif chemokine ligand 2(CCL2), also known as monocyte chemoattractant protein-1 (MCP-1). CCL2 is an important mediator of immune cell recruitment and is responsible for driving the chemotaxis of myeloid and lymphoid cells [38]. High CCL2 serum level is a prognostic marker for AML, with higher serum levels of this chemokine indicating poorer clinical outcomes in AML patients [39, 40]. We examined mRNA levels of CCL2 after treatment with MK256 in three AML cell lines, MOLM-14, MV4.11 and KG-1. The results showed that in MOLM-14 cells, when the cells were treated with MK256 at 300 nM, the mRNA level of CCL2 was approximately 5%; at 1 uM, the mRNA level of CCL2 was abolished (Figure 6C). We observed the similar significant inhibition of mRNA levels of CCL2 in MV4.11 cells after MK256 treatment (Figure 6D). In KG-1 cells, the inhibitory effect was less profound, but the expression of CCL2 was downregulated to approximately 50% and 40% with 300 nM and 1 uM of MK256, respectively (Figure 6E).

### *In vitro* ADMET, *in vivo* PK, PD, and toxicity study of MK256

In parallel to examining the mechanism of action (MOA) of MK256 in AML cell lines, we also evaluated its drug-like properties (Table 3). For this, we performed a set of *in vitro* ADMET assays. Kinetic solubility assay of MK256 showed a satisfactory solubility of 566 uM at pH 7.4. MK256 also had good cell permeability in the MDCK-MDR1 cell permeability assay, with an efflux ratio of 0.2. As shown in Table 3, *in vitro* metabolic stability of MK256 was tested in three different species. The half-life of MK256 in human, dog, and mouse was 89 min, 214 min and 311 min, respectively. Intrinsic clearance data for MK256 in the three species suggested a medium level of clearance of MK256 in all species. We also examined the possibility of drug-drug interaction of MK256 by testing it in a cytochrome P450 inhibition assay. Among the seven main cytochrome P450 isoforms (CYP1A2, CYR2C8, CYP2C9, CYP2C19, CYP2D6, CYP3A4-MDZ, CYP3A4-TS), MK256 showed no inhibition at the tested concentration of 50 uM. We also evaluated the cardiotoxic effect of MK256 by testing it at 30 uM in a hERG safety assay. Results indicated no inhibition of hERG at 30 uM. Collectively, our results suggest a desirable drug-like profile of MK256.

**Table 3:** Physicochemical property, *in vitro* ADME and *in vivo* PK of MK256

Solubility				
KSOL (μM) PBS (PH 7.4)		566		
**MDCK-MDR1 cell permeability**				
**Concentration (μM)**	**Papp, A-B (X10^−6^ cm/s)**	**Papp, B-A (X10^−6^ cm/s)**	**Efflux Ratio (B-A/A-B)**	**Recover rate (%)**
5	57	14	0.2	96
**Hepatocyte Stability**				
**Species**	**T_½_ (min)**	**Clint (μL·min^−1^·mg protein^−1^)**		
Human	89	16		
Dog	214	7		
Mouse	311	5		
***In vivo* mouse PK1 **				
C_max_^2^ (μM)		29		
AUC_last_ (hr * μM)^2^		330		
T_1/2_ (hr)^2^		3.6		
Cl (mL·hr^**−**1 ^·kg^**−**1 ^)		170		
V_ss_ (L/kg)		0.49		
F (%)		59%		

**Figure 7:**
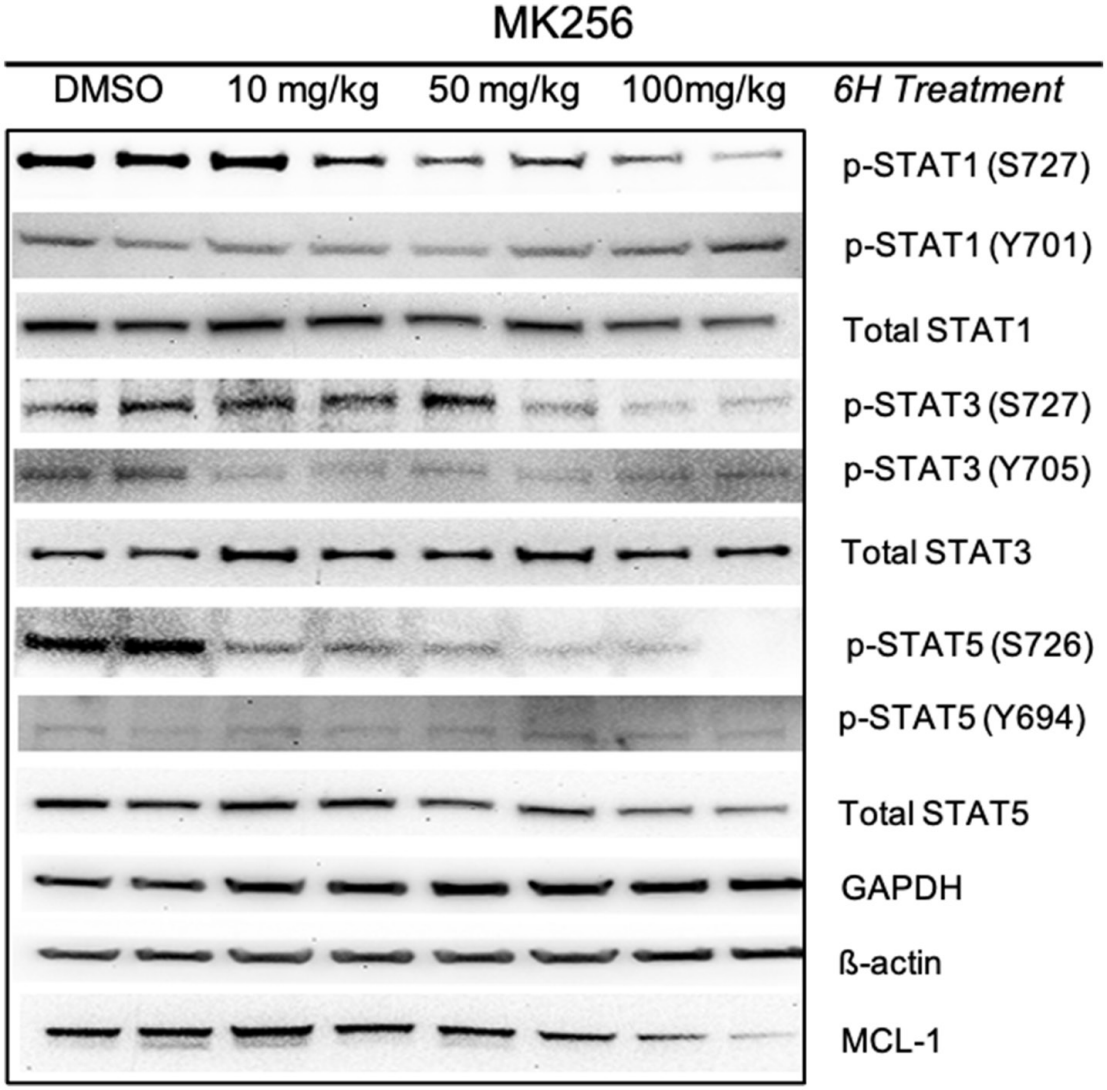
Pharmacodynamics study of MK256 in MOLM-14 xenograft model. MK256 was given orally to mice at 10 mg/kg, 50 mg/kg and 100 mg/kg.

**Figure 8:**
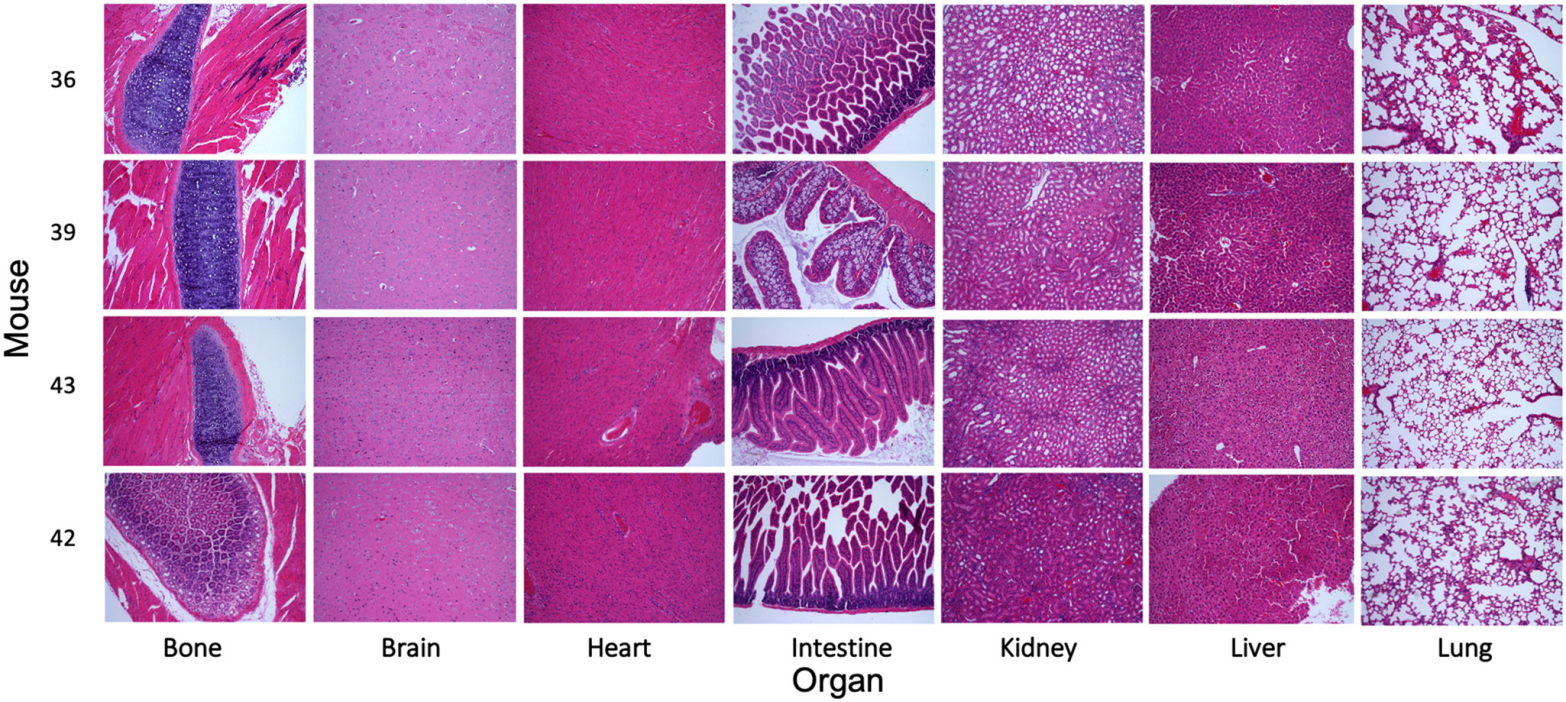
Evaluation of the toxicity of MK256 *in vivo*. Histological data (H&E staining) obtained in the major organs (bone, brain, heart, intestine, kidney, liver, and lung) of the mice treated with MK256 (100 mg/kg, q.d., oral) for 21 days.

To evaluate the behavior of MK256 *in vivo* we did a full pharmacokinetics (PK) analysis of MK256 in mice. The results showed that MK256 has a half-life of 3.6 hr and a bioavailability of 59% (Table 3). In the MOLM-14 xenograft model, we treated mice with 10 mg/kg, 50 mg/kg and 100 mg/kg of MK256 for 6 hrs, after which tumors from each treatment group were harvested and processed for western blotting analysis. The results showed that with higher doses of MK256, the expression levels of p-STAT1 (S727), p-STAT3(S727), p-STAT5(S726) were further downregulated (Figure 7). This suggests that MK256 can downregulate p-STAT1 (S727), p-STAT3(S727), p-STAT5(S726) in a dose-dependent manner *in vivo*. We also performed a histological analysis of the major organs (bone, brain, heart, intestine, kidney, liver and lung) of mice after they were treated with 100 mg/kg of MK256 for 21 days (q.d, oral) (Figure 8). The results indicated that prolonged and high-dose treatment of MK256 did not cause visible pathological changes in the organs, suggesting that there was no significant histological abnormality in the treated mice. In summary, MK256 offered a desirable solubility, permeability, hepatocyte stability, toxicology, and pharmacokinetics/pharmacodynamics (PK/PD) profile.

### MK256 showed great efficacy in MOLM-14 mice xenograft model

**Figure 9:**
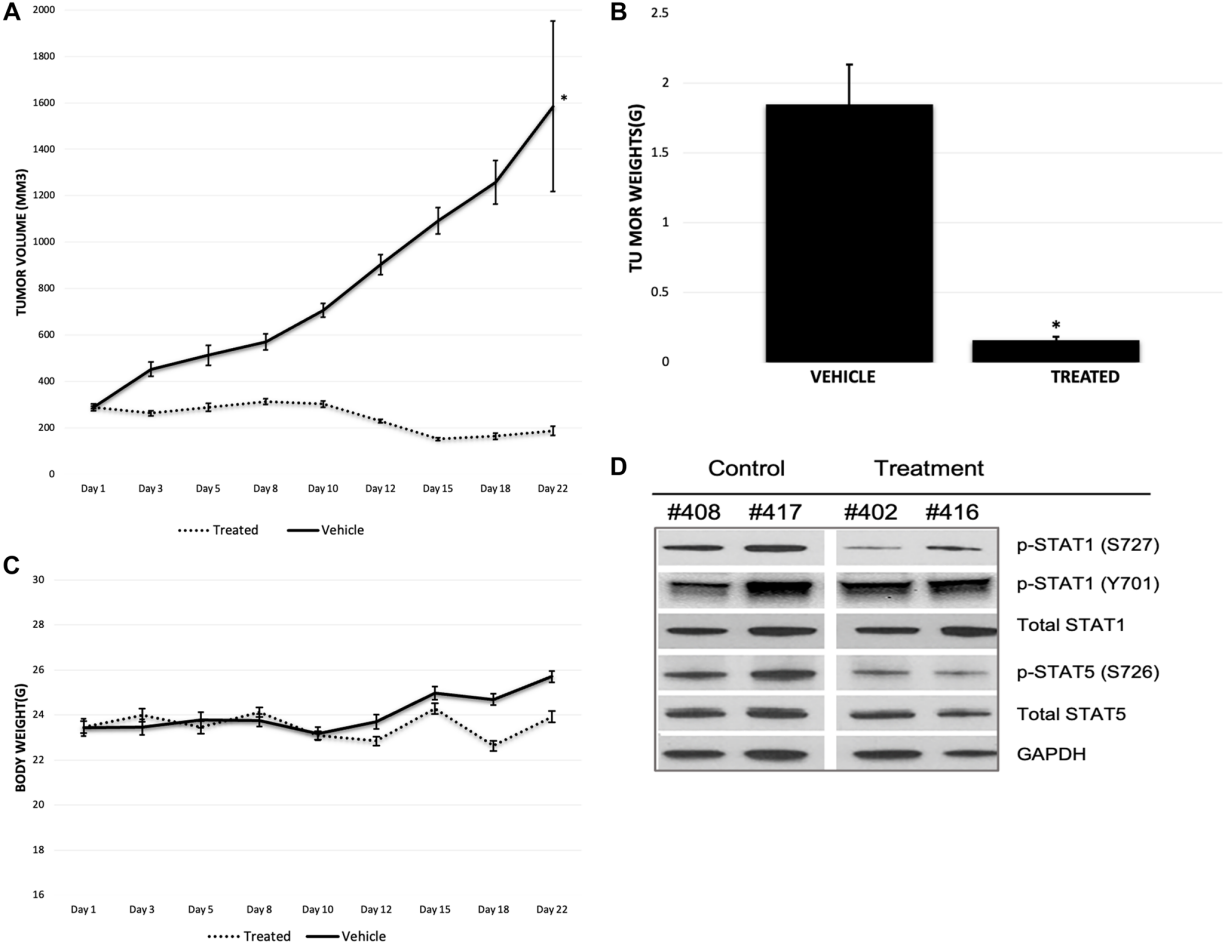
(**A**) Tumor growth of MOLM-14 xenograft model. Treatment group is represented with dashed line. Vehicle group is represented with straight line. ^*^ *P* < 0.05. (**B**) Tumor weight graph of MOLM-14 tumor model. ^*^ *P* < 0.05. (**C**) Body weight graph of MOLM-14 tumor model. Treatment group is represented with dashed line. Vehicle group is represented with straight line. (**D**) Western Blot of tumor samples of MOLM-14 tumor model. Mouse tag numbers from control (#408, #417) and treatment (#402, #416) were shown.

In our experiments, MK256 showed both potent inhibition and dose-dependent downregulation of the STAT pathway in multiple AML cell lines and exhibited a desirable PK/PD profile. These favorable properties led us to evaluate the efficacy of MK256 *in vivo* using the MOLM-14 cell line to examine if the compound could inhibit tumor growth in the MOLM-14 xenograft model. Mice were given 100 ul of 50 mg/kg of MK256 or 100 ul of saline twice a day for 21 days following a 5-day on/ 2-day off schedule. Tumors from two groups of mice were all initially ~ 300 mm^3^. Tumors of the mice in the control group continued to grow quickly during the 21-day period, with the average tumor volume reaching 1600 mm^3^ on day 22, when we measured tumors for the last time. In the contrast, tumors of the mice in the treatment group shrank to 200 mm^3^ on average by day 22 (Figure 9A). Average tumor weight was 0.16 g for the treatment group and 1.85 g for the control group (Figure 9B). These results suggests that MK256 can effectively inhibit the tumor growth in the MOLM-14 mice tumor model. Average body weight of mice increased from 23.4 g to 25.7 g in the control group and from 23.5 g to 23.9 g in the treatment group, suggesting that MK256 did not produce significant weight loss and was well-tolerated at the selected dosing regimen (Figure 9C). Tumors collected on day 22 were then processed and analyzed with western blot. The results indicated that MK256 can down regulate the expression level of p-STAT1(S727) and p-STAT5(S726) not only *in vitro* but also *in vivo* (Figure 9D).

### Combination treatment with MK256 and venetoclax in the MOLM-14 cell line *in vitro* and *in vivo*

**Figure 10:**
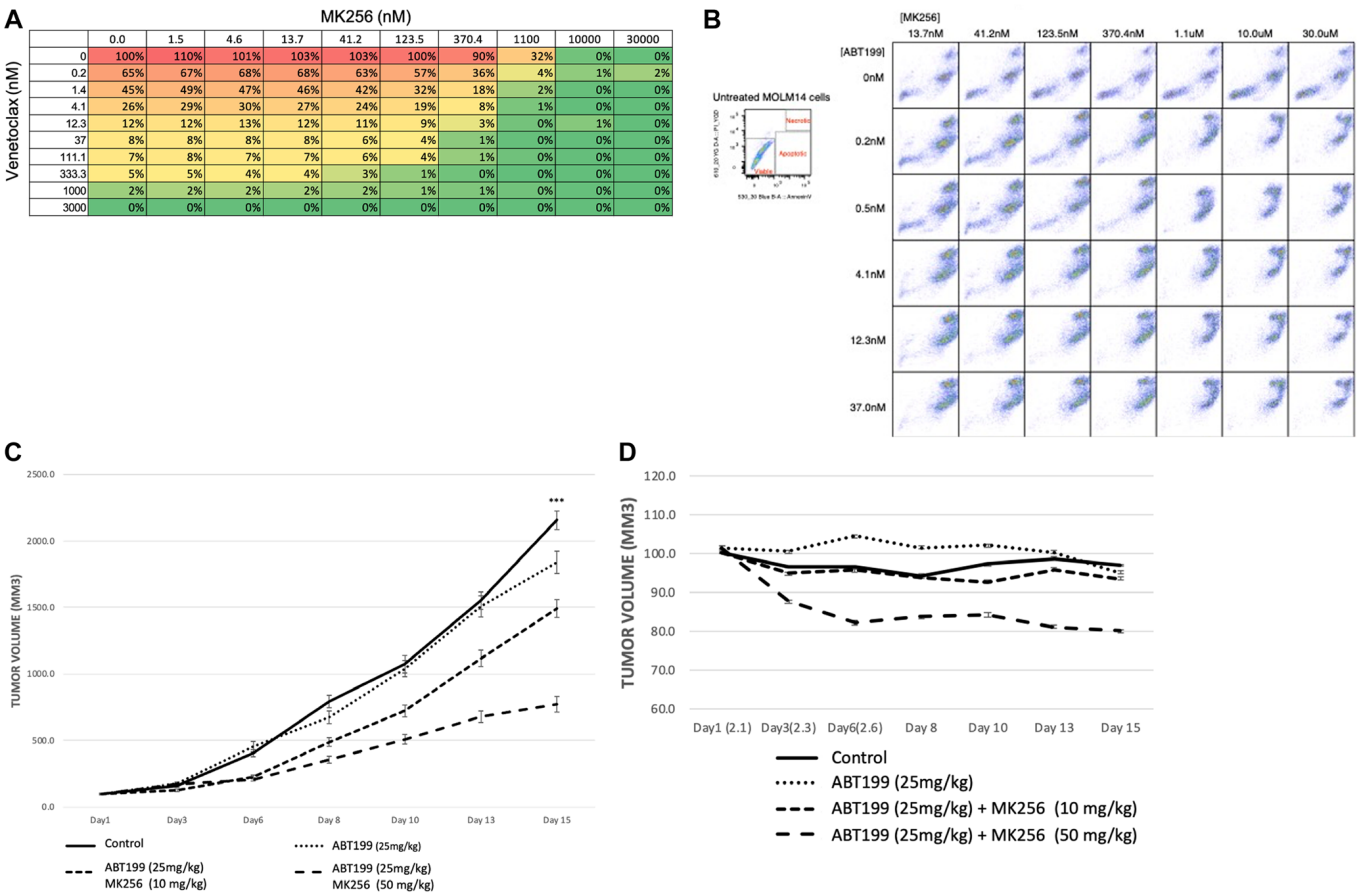
(**A**) Dose-response matrix of percentage of cell viability of 10,000 MOLM-14 cells after 72 hrs treatment of MK256 and venetoclax at various concentration combinations. (**B**) Annexin V staining of 10,000 MOLM-14 cells after 72 hrs treatment of MK256 and venetoclax(ABT199) at various concentration combinations. Status of cells are labeled in the graph at the upper left corner. (**C**) Tumor growth of MOLM-14 xenograft model. Groups of control; ABT199 (25 mg/kg); ABT199 (25 mg/kg) + MK256 (10 mg/kg); ABT199 25 mg/kg + MK256 (50 mg/kg) were shown in straight line, dotted line, 3 dashed line and 2 dashed line, respectively. ^***^ *P* < 0.001. (**D**) Body weight percentage of MOLM-14 tumor model.

Our earlier experiments showing the clearly increasing expression of BCL-2 in the MOLM-14 cell line led us to ask if there was any combinational effect between MK256 and a BCL-2 inhibitor in MOLM-14 cells. To address this question, we chose venetoclax (ABT-199), an FDA-approved BCL-2 inhibitor for AML and CLL, for the combination study. We treated MOLM-14 cells with MK256 and venetoclax at a series of concentration combinations, measured cell viability, and reported the data in a dose-response matrix (Figure 8). The combination of MK256 and venetoclax lowered the effective concentration for both compounds, resulting in additive effect in the cell line (Figure 10A). We also investigated the apoptotic effect of the combination treatment in the MOLM-14 cell line (Figure 10B). Flow cytometry showed a considerable fraction of viable cells was present when 10,000 MOLM-14 cells were treated with 30 uM MK256 for 72 hrs. However, at concentrations where MK256 is higher than 1.1 uM and venetoclax is higher than 0.2 nM, the viable cells started to disappear.

In an efficacy study of the combination of MK256 and venetoclax in the MOLM-14 tumor model, we compared venetoclax 25 mg/kg, venetoclax 25 mg/kg + MK256 10 mg/kg, and venetoclax 25 mg/kg + MK256 50 mg/kg. Compounds were given orally once a day on a 5 days on/2 days off schedule. In this study, venetoclax only inhibited the tumor growth by 15% if administered alone at 25 mg/kg. Combination treatment with 10 mg/kg MK256 enhanced the efficacy of venetoclax to 33% inhibition, whereas 50 mg/kg MK256 enhanced the efficacy of venetoclax to 70% inhibition (Figure 10C). However, mice treated with venetoclax 25 mg/kg + MK256 50 mg/kg had a 20% weight loss (Figure 10D). Nonetheless, these collective findings suggest that MK256 and venetoclax can have additive effects in the MOLM-14 xenograft model, and potential as a combination to investigate in clinical trials.

## DISCUSSION

CDK8 provides novel opportunities for the development of cancer therapeutics. It has been associated with different types of cancers including AML, ALL [41, 42], colorectal cancer [43–45], melanoma [46], breast cancer [47], pancreatic cancer [48, 49], and glioblastoma [50]. To the best of our knowledge, the most advanced CDK8 inhibitor is SEL120, which is being investigated in phase I studies for the treatment of AML and High-risk Myelodysplastic Syndrome (HR-MDS) (NCT04021368) [29]. Cortistatin A (CA) is another highly potent natural product CDK8 inhibitor [41]. Pelish et al. found that CA selectively inhibited AML cell lines *in vitro* and *in vivo*, and that CA upregulated tumor suppressor and lineage-controlling genes associated with super-enhancer in the sensitive AML cell lines [41]. However, the inhibitory effect of CA on the STAT pathway of CA was not examined. Despite the promising effects showing by CDK8 inhibitors, Clarke et al. argued that major systemic toxicities observed in rats and dogs after treatment with two chemically distinct prototype CDK8 inhibitors—Cmpd3 and Cmpd4— were on-target effects, implying the invalidity of CDK8 as a therapeutic target [51]. However, Chen et al. used three other CDK8 inhibitors—senexin B, dCA and 15W—to demonstrate that no such toxicities were linked to CDK8 inhibition and to argue that the toxicity of Cmpd3 and Cmpd4 may be induced by significant off-target inhibition, as shown by kenome profiling and Kd determination [52]. Cmpd3 was found by Chen et al. to significantly inhibit PIKFYVE, JNK1, and STK16 and Cmpd4 to GSB3B and GSB3A [52].

Targeting CDK8 may be a promising strategy for preventing drug resistance. Sharko et al. found that the CDK8/19 inhibitors senexin B and 15W prevented resistance to gefitinib and erlotinib in breast cancer cell lines BT474 and SKBR3 [53]. The same effect was observed in the SW48 colorectal cell line in which senexin B prevented the development of cetuximab resistance [53]. The work by by Sharko et al. indicates the effect of CDK8 inhibition on the development of resistance and suggests potential use of CDK8 inhibitors in preventing resistance to current drugs. Since MK256 is a more potent CDK8 inhibitor than senexin B and 15W, whether it prevents the development of resistance in the same context is an area for further study.

More interestingly, CDK8 inhibitors may have the potential to be effective immune modulators for various cancers. Natural killer (NK) cells are the first line responders which defend against tumor cells. In a study by Witalisz-Siepracka et al., deletion of CDK8 enhanced the NK-cell cytotoxicity through releasing more perforin *in vitro*, and enhanced cytotoxicity was apparent in three *in vivo* models: B16F10 melanoma, v-abl+ lymphoma, and an oncogene-driven leukemia [54]. These findings indicated a suppressive role of CDK8 in NK cell function. Therefore, it is reasonable to consider that CDK8 inhibitors might kill tumors through enhancing NK cell responses. The potential of MK256 as an NK cell enhancer should be investigated in future studies.

In summary, our study shows that MK256, a potent and selective CDK8 inhibitor, is an effective negative STAT pathway regulator both *in vitro* and *in vivo* in AML. We observed satisfying efficacy of MK256 in the MOLM-14 xenograft model. The preclinical success of this compound demonstrates its potential to target LSCs in AML to prevent disease relapse and improve overall survival.

## MATERIALS AND METHODS

### Kinase profiling

MK256 was synthesized in house. The kinase profiling of MK256 against various targets was done using the SelectScreen Kinase Profiling Services provided by ThermoFisher Scientific (Walham WA, USA). https://www.thermofisher.com/us/en/home/products-and-services/services/custom-services/screening-and-profiling-services/selectscreen-profiling-service/selectscreen-kinase-profiling-service.html.

### Docking and molecular dynamics simulation of MK256 in CDK8

Discovery Studio 2020 Client (DS2020, Biovia, San Diego, CA, USA) was used to dock MK256 into the ATP binding site of CDK8. The structure of CDK8 used for docking was prepared from the X-ray crystal complex of CDK8/cyclin C and compound 109 (PDB: 5HBJ, resolution: 3.00 Å). To prepare the structure for docking: 1) Cyclin C and water molecules were removed from the crystal complex; 2) the space occupied by the previously bound inhibitor compound 109 was recognized by DS2020 as the binding site for MK256; 3) MK256 was prepared by Prepare Ligands in DS2020 to assign 3D coordinates, biological ionization and tautomerization states. After the protein and ligand were prepared, docking protocol LibDock in DS2020 was used for docking. Default parameters of LibDock were unchanged. Docked poses of MK256 were examined after LibDock docking. Poses exhibiting H-bonds formed between hinge region and MK256 were paid special attention. LibDock scores of poses were ranked. In the end, the best possible pose was chosen as the one with the highest LibDock score and reasonable H-bonds with hinge region. Then the pose was subjected to molecular dynamics (MD) simulation. The CDK8/MK256 protein structure (PDB: 5HBJ) was assigned with charmm36. The Solvation protocol was used to solvate the structure of CDK8/MK256. The complex was placed in an orthorhombic box with periodic boundary conditions containing 10514 water molecules. The radius of explicit solvent is 20 Å. Counterions, 28 sodium, and 38 chloride were added to neutralize the system to reach a final concentration of 145 mM. MK256 was deprotonated at carboxylic acid moiety and assigned a -1 charge. The MD simulation was devised into four subsequential steps. In the minimization step, 4000 steps of Steepest Descent followed by 6000 steps of Conjugate Gradient were applied to the system to remove any clash or unfavorable confirmations. The system was then heated by 10 ps to reach a target temperature of 300K. In the equilibration step, the system was equilibrated by 100 ps when potential energy of the complex reached a steady state. MD simulation was performed using NPT schedule with T = 300K and P = 1 bar. The simulation time step was 2 fs. The total simulation time was 1 ns.

### Kinetic solubility assay

MK256 was supplied as DMSO stock at 10 mM. 190 μL of buffer solution (PBS, pH 7.4) was added to all wells on a 96-well Millipore Solubility filter plate. 10 μL of MK256 or control compound testosterone was transferred in triplicate to the buffer wells to a final concentration of 500 μM. The filter plate was shaken for 1.5 hours at room temperature. Samples were filtered via a vacuum system into a fresh 96-well plate. Compounds were diluted to 500 μM (highest concentration) in DMSO and further diluted 1:10 for calibration curve (three-point). HPLC/UV analysis was performed on all samples (220 nm, 254 nm, and 280 nm).

### MDCK-MDR1 permeability assay

MDCK-MDR1 cells were plated into 96-well Millipore Millicell-96 plates at 7,500 cells/75 μL/well and incubated for three days at 37°C with 5% CO2. Cells were washed with Hank’s Balanced Salt Solution (HBSS) with 5 mM HEPES for 30 minutes before the experiment began. MK256 was prepared by diluting DMSO stock into HBSS buffer, resulting in a final DMSO concentration of 0.1%. Prior to the experiment, cell monolayer integrity was verified by transendothelial electrical resistance (TEER). The transport experiment was initiated by adding MK256 to the apical (75 μL) or basal (250 μL) side. Transport plates were incubated at 37°C in a humidified incubator with 5% CO2. Samples were taken from the donor and acceptor compartments after one hour and analyzed by liquid chromatography with tandem mass spectrometry (LC/MS/MS). Digoxin was used as reference control. Apparent permeability (Papp) values were calculated using the following equation: Papp = (dQ/dt)/A/C_0_, where dQ/dt is the initial rate of amount of test compound transported across the cell monolayer, A is the surface area of the filter membrane, and C_0_ is the initial concentration of the test compound, calculated for each direction using a 4-point calibration curve by LC/MS/MS. Net flux ratio between the two directional transports was calculated by the following equation: Ratio = Papp, B-A/Papp, A-B, where Papp, B-A and Papp, A-B represent the apparent permeability of test compound from the basal-to-apical and apical-to-basal side of the cellular monolayer, respectively. Recovery was calculated based on the compound concentration at the end of the experiment, compared to that at the beginning of the experiment, adjusted for volumes.

### Hepatocyte stability assay

Human LiverPoolTM 20-donor, dog and mouse cryopreserved hepatocytes were obtained from BioreclamationIVT. Cryopreserved hepatocytes were removed from the liquid nitrogen tank and thawed in a 37°C water bath. As soon as the cells pulled away from the vial wall, they were decanted into 48 ml of warm HT medium. Cells were centrifuged for four minutes at 420 rpm (50 g). After the supernatant was removed, the pellet was re-suspended in warm DMEM medium. Cell density was counted by a hemacytometer. The assay was carried out in 96-well microtiter plates. MK256 was incubated for 0, 60, 120, and 180 minutes at 37°C with hepatocytes. Reaction mixtures (50 μL) contained a final concentration of 1 μM MK256, 0.5 million cells/mL hepatocytes in the DMEM medium. At each time point (for example, 0, 1, 2, and 3 hrs), 200 μL of quench solution (100% acetonitrile with 0.1% formic acid) with internal standard were transferred to each well. Midazolam was included as a positive control to verify assay performance. Plates were sealed and centrifuged at 4°C for 15 minutes at 4000 rpm. The supernatant was transferred to fresh plates for LC/MS/MS analysis. All samples were analyzed on LC/MS/MS using an AB Sciex API 4000 instrument, coupled to a Shimadzu LC-20AD LC Pump system. Analytical samples were separated using a Waters Atlantis T3 dC18 reverse phase HPLC column (20 mm × 2.1 mm) at a flow rate of 0.5 mL/min. The mobile phase consisted of 0.1% formic acid in water (solvent A) and 0.1% formic acid in 100% acetonitrile (solvent B). The extent of metabolism was calculated as the disappearance of the test compound, compared to the 0-min control reaction incubations. Initial rates were calculated for the compound concentration and used to determine t1/2 values and subsequently, the intrinsic clearance, CLint = (0.693)(1/t1/2 (min))(mL incubation/million cells).

### *In vivo* PK study

Mice were dosed with either 1 mg/ml (IV) at a dose of 3 mg/kg or 3 mg/ml (PO) at a dose of 30 mg/kg in 40% PEG400 + 60% (30% Solutol) (final pH ~ 7). Blood samples were drawn at 5 mins, 15 mins, 30 mins, 1 hr, 2 hrs, 4 hrs, 8 hrs, and 24 hs from mice dosed with the IV route and at 1 hr, 4 hrs, 8 hrs and 24 hrs from mice dosed orally. An aliquot of 30 μL of plasma was extracted with 100 μL of 5:95 methanol:acetonitrile containing internal standard (Verapamil). The mixture was vortexed on a shaker for 15 minutes and subsequently centrifuged at 4000 rpm for 15 minutes. An aliquot of 70 μL of the supernatant was mixed with 70 μL water with 0.1% formic acid for the injection to the LC/MS/MS. Calibration standards were prepared by spiking MK256 into the blank mouse plasma. All samples are analyzed on LC/MS/MS using an AB Sciex API 4000 instrument, coupled to a Shimadzu LC-20AD LC Pump system.

### Cell viability assay

AML cell lines MV-4-11, MOLM14, KG-1, and SKNO-1 were acquired from ATCC (Manassas, VA, USA) and DSMZ (Braunschweig, Germany). Cells were plated (96-well) in triplicate at 3000 cells per well for testing (*n* = 3) in 1% FBS media. On day 7, the CellTiter-Glo Luminescent Cell Viability Assay (Promega, Madison, WI, US) was performed to measure the response. Two independent experiments were done.

### Real-time PCR

Total RNA from the various cell lines was isolated using the RNeasy extraction method (Qiagen, Valencia, CA, USA). First-strand cDNA was synthesized from total RNA by iScript cDNA synthesis (Bio-Rad, Hercules, CA, USA) according to the manufacturer’s instructions. Taqman RT–PCR analysis was performed on cDNA in a 384-well plate, using a Prism 7900HT Real-Time PCR System (Applied Biosystems, Foster City, CA, USA). Primers and Taqman probes were purchased from Applied Biosystems. The expression of target genes MCL1 (Hs03043899_m1, Thermo fisher) and CCL2 (Hs00234140_m1, Thermo fisher) in each sample was assayed in triplicate and normalized to human GUSB for mRNA expression analysis. An unpaired t test was used to calculate statistical significance.

### Western blot

For the *in vitro* western blot study, MV-4-11 and MOLM-14 cells were plated (6-well) at 5 × 10^5^ cells per well with medium containing 1% FBS overnight. MK-256 was added in different concentrations at different time points. After 16 hours, cells were lysed in M-per Mammalian Protein Extraction Reagent (78501, Thermo Fisher Scientific) containing protease and phosphatase inhibitors (88669, Thermo Fisher Pierce). Total protein concentration was measured using the Pierce BCA protein Assay Kit (23227, Thermo Fisher Scientific). Proteins were separated on SDS-PAGE and transferred to Nitrocellulose membrane.

For the *in vivo* western blot study, to assess gene expression *in vivo*, tumors were induced in female NOD SCID mice by s.c. inoculation of 5 × 10^6^ AML cells suspended in matrigel. Mice bearing tumors of at least 100 mm^3^ were treated with MK256. Mice were sacrificed after 6 hrs and tumors were harvested. The tumors were homogenized and lysed in T-per Mammalian Protein Extraction Reagent (78510, Thermo Fisher Scientific) containing protease and phosphatase inhibitors (88669, Thermo Fisher Scientific). Total protein concentration was measured using the Pierce BCA protein Assay Kit (23227, Thermo Fisher Scientific). Proteins were separated on SDS-PAGE and transferred to Nitrocellulose membrane. Immunoblotting was performed using specific antibodies. The following specific antibodies were used: Cell signaling technology: pSTAT1(S727) #8826, pSTAT1(Y701) #9167, STAT1 #14994, pSTAT3 (S727) #9134, pSTAT3(Y705) #9145, STAT3 #9139, pSTAT5(Y694) #4322, STAT5 #25656, b-ACTIN #3700, GAPDH #5174, BCL2 #4223, MCL-1 #94296, Cleaved PARP #9541, Cleaved Caspase3 #9661, IL6ST #12859, SmyD3 #3732, PD-L1 #13684. Invitrogen: IL6R PA5109855. Abcam: pSTAT5(S726) ab128896.

### Flow cytometry

Healthy novel human leukemic cell line (TEX) cells were plated on 100 mm culture dishes at 5 × 10^5^ per well, starved with medium containing IMDM supplemented with 1.5%FCS, 2 ng/ml SCF, and 0.2 ng/ml IL-3 (Amgen, Thousand Oaks, CA, USA) overnight [30]. MK256 was added to final concentrations of 50 mM and 500 mM; the same condition applied to SEL-120, and with DMSO as negative control for 6-day treatments. Media with tested drugs was replaced on the third day. Following the 6-day treatments, cultures were washed once in Sort buffer (PBS + 2% FBS + 2 mM EDTA), and resuspended to a final concentration of 50 million cells/mL into a 96-well V-shaped plate. Cells were subsequently incubated with Zombie Aqua Fixable Viability Dye (Thermo), washed with Sort buffer, and incubated with Human FcX (BioLegend) to prevent non-specific antibody binding. Following FcX incubation, cells were washed with Sort buffer and incubated with cell surface antibody mix diluted in the BV stain buffer (BD Biosciences) following manufacturer instructions for 30 mins on ice in the dark. Cells were then washed with 100 mL Sort buffer and permeabilized in fresh Fix/Perm solution (Foxp3 Transcription Factor Staining Buffer from eBioscience) following the manufacturer’s instructions. Cell were subsequently incubated in intracellular stain mix in Perm Buffer (1 mL 10× Perm Buffer (eBiosceince) + 8.8 mL dH_2_O + 2% FCS) for 30 mins on ice in dark. Isotype controls were stained with mouse IgG conjugated to FITC, PE, PerCP-Cy5.5, PE-Cy7, APC, and Alexa Fluor™ 700. Antibodies used to stain cells were FITC-conjugated anti-CD93, PE-conjugated anti-CD96, PerCP-Cy5.5-conjugated anti-CD38, PE-Cy7-conjugated anti CD-34, APC-conjugated anti-CD117, and Alexa Fluor™ 700-conjugated LIVE/DEAD (Invitrogen). Stained samples were resuspended in Perm Buffer and run on a FACSAriaII Flow Cytometer (BD Biosciences). Data were analyzed in FACSDiva (BD Biosciences) and FlowJo (Treestar). To determine the absolute numbers of cells in samples analyzed by FACS, AccuCount beads (Spherotech) were added to stained samples prior to each FACS run per manufacturer’s instructions, and absolute cell counts were calculated based on the known number of beads added. In order to cellularly differentiate apoptosis in AML cells, MOLM14, as a result of synergetic treatments of MK256 combined with ABT199, we used Dead Cell Apoptosis Kits with Annexin V (Invitrogen). MOLM14 cells were plated on a 96-well V-shaped plate at 5x105 per well, starved with medium containing 99% RPMI 1640, 1% FBS, and 1X Antibiotic-Antimycotic (Invitrogen). MK256 were added to final concentrations of 13.7 nM, 41.2 nM, 123.5 nM, 370.4 nM, 1.1 mM, 10.0 mM mM on each column, while ABT199 was at final concentrations of 0.2 nM, 0.5 nM, 4.1 nM, 12.3 nM, 37.0 nM on each row. Following 72-hr retreatment, cultures were washed once in Sort buffer (PBS + 2% FBS + 2 mM EDTA) and resuspended to a final concentration of 10^4^ cells/well into a 96-well V-shaped plate. Cells were subsequently incubated with Alex Fluor™ 488 annexin V and PI for Flow Cytometry following manufacturer’s instructions.

### *In vivo* mouse study

Female NOD SCID mice were purchased from Charles River Laboratories (Wilmington, USA) or bred at the animal facilities of the University of California San Francisco. All animal experiments were carried out under protocols approved by the Institutional Animal Care and Use Committee of the University of California San Francisco. For MOLM-14 model, 200 ul of 1 × 10^6^ cells combined with matrigel (v/v, 1:1) were injected subcutaneously in the right flank of NODSCID mice. Tumors were allowed to grow for two weeks to reach a tumor volume about 200 mm^3^ before mice were randomly grouped. The animals were treated for three weeks on a 5 days on/2 days off schedule by oral gavage with MK256 (dissolved in saline) alone or in combination with Venetoclax (dissolved in 5% DMSO + 50% PEG 300 + 5% Tween 80 + ddH_2_O). In the single treatment study, MK256 was administered by mice twice a day at 50 mg/kg. In the combination study, MK256 (10 or 50 mg/kg) and was coadministered with Venetoclax (25 mg/kg) once a day. Tumor volume and body weight were monitored once every 3 days. Tumor volume was calculated as L × W^2^/2, where L and W were the length and width of the tumor, respectively.

## Abbreviations

AML: acute myeloid leukemia
CDK8: Cyclin dependent kinase 8
LSCs: Leukemic stem cells
HSCs: hematopoietic stem cells
JAK/STAT: Janus kinase/signal transducers and activators of transcription
p-STATs: phosphorylated STATs
CTD: c-terminal domain
TGF-β: transforming growth factor-β
BMP: bone morphogenetic protein
SEL120: SEL120-34A
CADD: computer-aided drug design
TEX: novel human leukemic cell line
HPSC: hematopoietic stem cell
ALL: acute lymphoid leukemia
MLL: mixed lineage leukemia
CCL2: C-C motif chemokine ligand 2
MCP-2: monocyte chemoattractant protein-1
MOA: mechanism of action
PK/PD: pharmacokinetics/Pharmacodynamics
HR-MDS: High-risk Myelodysplastic Syndrome
NK: Natural killer cells
DS2020: Discovery Studio 2020 Client
MD: molecular dynamics
HBSS: Hank’s Balanced Salt Solution
TEER: transendothelial electrical resistance
LC/MS/MS: liquid chromatography with tandem mass spectrometry
Papp: Apparent permeability
